# Optical Coherence Tomography Angiography (OCT-A) in Uveitis: A Literature Review and a Reassessment of Its Real Role

**DOI:** 10.3390/diagnostics13040601

**Published:** 2023-02-07

**Authors:** Carl P. Herbort, Masaru Takeuchi, Ioannis Papasavvas, Ilknur Tugal-Tutkun, Alireza Hedayatfar, Yoshihiko Usui, Pinar C. Ozdal, Cristhian A. Urzua

**Affiliations:** 1 Retinal and Inflammatory Eye Diseases, Center for Ophthalmic Specialised Care (COS), Rue Charles-Monnard 6, 1003 Lausanne, Switzerland; 2 Department of Ophthalmology, National Defence Medical College, Tokorozawa 258-8513, Japan; 3 Department of Ophthalmology, Istanbul Faculty of Medicine, Istanbul University, Istanbul 34093, Turkey; 4 Eye Research Center, The Five Senses Institute, Rassoul Akram Hospital, Iran University of Medical Sciences, Tehran 14535, Iran; 5 Noor Ophthalmology Research Center, Noor Eye Hospital, Tehran 14535, Iran; 6 Department of Ophthalmology, Tokyo Medical University Hospital, Tokyo 160-8402, Japan; 7 Department of Ophthalmology, University of Health Sciences, Ankara 98901, Turkey; 8 Laboratory of Ocular and Systemic Autoimmune Diseases, Faculty of Medicine, University of Chile, Santiago 1058, Chile; 9 Facultad de Medicina, Clinica Alemana-Universidad del Desarrollo, Santiago 1058, Chile

**Keywords:** optical coherence tomography angiography (OCT-A), dual fluorescein angiography (FA) and indocyanine green angiography (ICGA), posterior uveitis, dye imaging methods, multiple evanescent white dot syndrome (MEWDS), acute posterior multifocal pigment epitheliopathy (APMPPE), idiopathic multifocal choroiditis (MFC)

## Abstract

Background: The global and precise follow-up of uveitis has become possible with the availability of dual fluorescein (FA) and indocyanine green angiography (ICGA) since the mid-1990s. Progressively, additional non-invasive imaging methods have emerged, bringing value-added precision to the imaging appraisal of uveitis, including, among others, optical coherence tomography (OCT), enhanced-depth imaging OCT (EDI-OCT) and blue light fundus autofluorescence (BAF). More recently, another complementary imaging method, OCT-angiography (OCT-A), further allowed retinal and choroidal circulation to be imaged without the need for dye injection. Purpose: The purpose of this review was aimed at examining the evidence in published reports indicating whether OCT-A could possibly replace dye angiographic methods, as well as the real practical impact of OCT-A. Methods: A literature search in the PubMed database was performed using the terms OCT-angiography and uveitis, OCTA and uveitis and OCT-A and uveitis. Case reports were excluded. Articles were classified into technical reports, research reports and reviews. Articles in the two latter categories were analyzed in a more detailed, individual fashion. Special attention was paid to whether there were arguments in favor of an exclusive rather than complementary use of OCT-A. Furthermore, a synthesis of the main practical applications of OCT-A in the management of uveitis was attempted. Results: Between 2016 (the year of the first articles) and 2022, 144 articles containing the search terms were identified. After excluding case report articles, 114 articles were retained: 4 in 2016, 17 in 2017, 14 in 2018, 21 in 2019, 14 in 2020, 18 in 2021 and 26 in 2022. Seven articles contained technical information or consensus-based terminology. Ninety-two articles could be considered as clinical research articles. Of those, only two hinted in their conclusions that OCT-A could hypothetically replace dye methods. The terms mostly used to qualify the contribution of the articles in this group were “complementary to dye methods”, “adjunct”, “supplementing” and other similar terms. Fifteen articles were reviews, none of which hinted that OCT-A could replace dye methods. The situations where OCT-A represented a significant practical contribution to the practical appraisal of uveitis were identified. Conclusion: To date, no evidence was found in the literature that OCT-A can replace the classic dye methods; however, it can complement them. Promoting the possibility that non-invasive OCT-A can substitute the invasive dye methods is deleterious, giving the elusive impression that dye methods are no longer inevitable for evaluating uveitis patients. Nevertheless, OCT-A is a precious tool in uveitis research.

## 1. Introduction and Brief Overview of the Development of Imaging in Uveitis

Imaging plays a crucial role in the appraisal of uveitis, especially posterior uveitis. Since the development of fluorescein angiography (FA) by Novotny and Alvis in the early 1960s [1], constant progress occurred with the advent of ever-new modalities, starting with indocyanine green angiography (ICGA) and the publication of its schematic interpretation in uveitis [2]. This was followed by non-invasive methods, including, to cite only the most currently used modalities, optical coherence tomography (OCT), blue light fundus autofluorescence (BAF), enhanced depth imaging OCT (EDI-OCT) and, finally, OCT-angiography (OCT-A), adding more precision to the investigation of posterior uveitis.

### 1.1. Dual Fluorescein (FA) and Indocyanine Green Angiography (ICGA)

The global and precise follow-up of posterior uveitis has become possible with the advent of dual FA and ICGA since the mid-1990s [3,4,5]. Precise and global monitoring of both retinal and choroidal inflammation could thus be achieved in current uveitis practice [6]. Moreover, dual FA/ICGA allowed the clinicians to quantitatively establish the degree of inflammation independently in both the retinal and the choroidal compartments, thanks to a numerical angiographic score at presentation and during follow-up [7]. This represented a considerable advantage over classical “standardization of uveitis nomenclature (SUN)” that promoted merely qualitative and inadequate vitreous haze estimation as the sole measurement of posterior uveitis. Although it was ignored by SUN, dual FA/ICGA rapidly became the gold standard in the diagnosis and management of uveitis as it gave global information on all the structures of the central and peripheral fundus. Dual FA/ICGA was also increasingly used in clinical uveitis trials [8,9]. The major drawback of FA/ICGA was the fact that injection of dyes was necessary, limiting the frequency at which the procedure could be performed.

FA/ICGA dual angiography was followed by complementary imaging modalities such as OCT, EDI-OCT and BAF, which contributed additional information in certain specific diseases but were unsuited to and did not pretend to replace angiographic methods. Thanks to their advantage of being non-invasive, they could easily be repeated, allowing for closer follow-up of the parameters they were able to measure.

### 1.2. Optical Coherence Tomography (OCT)

Among the new main imaging modalities complementary to dye angiography that were added to the arsenal of the uveitis specialist, OCT represented a major imaging progress. It uses rays of low-coherence light to obtain cross-sectional images of the retina. It provides information on the quasi-histological quality on the different layers of the retina in the macular and peri-macular areas, as well as on the retinal thickness. Therefore, it represented a valuable supplementary tool in the appraisal of posterior uveitis. OCT was found to be useful in the diagnosis and the observation of the evolution of inflammatory cystoid macular oedema (CMO), demonstrating, more effectively than FA, the axial distribution of the fluid [10]. The main practical advantage of OCT over FA was the possibility of closer follow-up of CMO and better fine-tuning of treatment without the need for dye injection. In practice, what is presently considered and suggested as the standard of care for the assessment of retinal inflammation is a baseline FA and OCT. The CMO and posterior pole pathologies can be further assessed closely and non-invasively by follow-up OCTs. An example of the precise morphological information furnished by OCT is a detailed demonstration of the ellipsoid zone and outer retina ischemic disruption in choriocapillaritis entities, including acute posterior multifocal placoid pigment epitheliopathy (APMPPE) and multiple evanescent white dot syndrome (MEWDS) secondary to inflammatory choriocapillaris non-perfusion [11,12]. Furthermore, the advancement in the technology of anterior segment OCT is promising for visualizing the anterior chamber and anterior vitreous in patients with anterior uveitis and anterior vitritis, using laser flare photometry in the evaluation of intraocular anterior inflammation [13].

### 1.3. Enhanced Depth Imaging OCT (EDI-OCT)

Despite the utility of conventional OCT to provide high-resolution cross-sectional images of the retina, the near-infrared light source used in many traditional OCT systems is scattered by the photoreceptor and retinal pigment epithelium layer, limiting its capability to visualize the underlying choroidal structures precisely. To overcome this limitation, some modifications in collecting the images of spectral domain OCT were applied to develop EDI-OCT [14] (swept-source or not), which provided more information on the choroidal compartment and allowed its thickness to be measured. Fluctuation of choroidal thickness on EDI-OCT occurs in case of stromal choroiditis, such as Vogt–Koyanagi–Harada disease (VKH) and HLA-A29 birdshot retinochoroiditis. Due to its non-invasive nature, repeated EDI-OCT imaging makes it possible to follow these conditions more closely and frequently, an advantage which ICGA, despite being the gold standard exam for such situations, does not possess [15]. Moreover, EDI-OCT, contrary to ICGA, can provide accurate three-dimensional images of the choroidal layers with precise measurement of the depth and morphology of the lesions.

### 1.4. Blue Light Fundus Autofluorescence (BAF)

**Figure 1:**
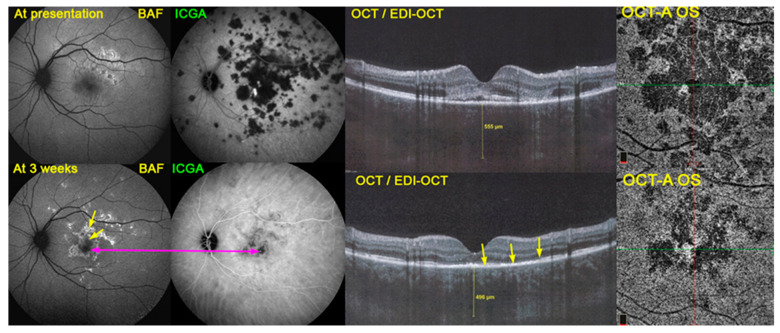
Multimodal imaging in APMPPE combining BAF, ICGA and OCT/EDI-OCT. This combination of invasive (ICGA) and non-invasive methods (BAF and OCT/EDI-OCT) gives a precise appraisal of the condition. BAF at presentation (**top left**) still showed minimal changes despite the extensive choriocapillary non-perfusion shown on ICGA (**top middle**), because the overlying RPE and outer retina were still at an edematous stage, as shown on OCT/EDI-OCT (**top right**). Three weeks later, after the partial re-perfusion seen on ICGA (**bottom middle**), the damaged areas were visible on BAF (**bottom left**). In the center, there was hypoautofluorescence corresponding to remaining non-perfusion on ICGA (crimson arrow). The bright hyperautofluorescent areas correspond to both suffering RPE cells with accumulated fluorophores and to the loss of photoreceptor outer segments, as shown on OCT/EDI-OCT (**bottom right**) (yellow arrows). OCT-A did not yield additional information, showing the same non-perfusion areas as seen on ICGA (choriocapillary drop out), limited to the central macular area, but was useful in the follow-up of these central damaged areas (also see Figure 3). (Far-right) OCT-A images at presentation (**top right**) and after 3 weeks (**bottom right**).

BAF is a valuable tool which is becoming more and more frequently used in daily practice by ophthalmologists. It does not require the injection of a dye in order to image the retina, but instead, it takes advantage of the (auto)fluorescent properties of lipofuscin within the RPE to provide information on pathologic changes in the fundus. As for other modalities, it is hampered by artifacts from media opacities such as cataracts or vitreous opacities, making the interpretation of images in some patients difficult. BAF can provide information on the RPE and the outer retina, and in some cases, can replace the dye exams involved in the follow-up of certain uveitis entities. Hypoautofluorescence indicates loss of RPE due to atrophy or scarring. Hyperautofluorescence can indicate damage to the RPE cells unable to eliminate fluorophores that accumulate unduly. Another explanation of hyperautofluorescence is the damage to the photoreceptor’s outer segments, unveiling the normal auto-fluorescent RPE lipofuscin due to the loss of the normal screen of the photoreceptors. The disease entity that can present all three situations simultaneously is APMPPE [16,17], (Figure 1).

**Figure 2:**
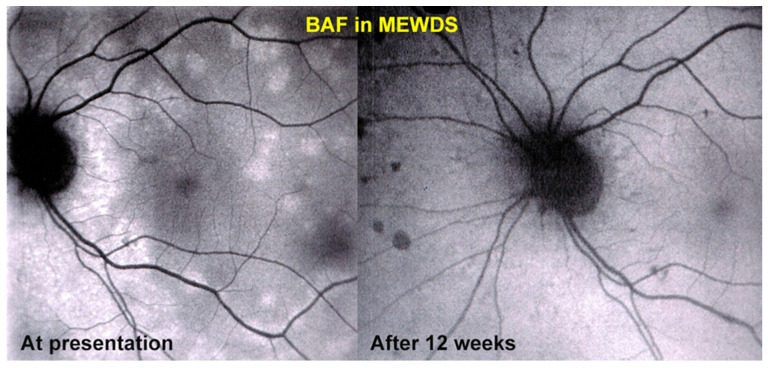
BAF in choriocapillaritis. BAF is of comparable value to ICGA to follow choriocapillaritis entities such as MEWDS or MFC. This patient was diagnosed and followed as MEWDS thanks to BAF. At presentation, numerous areas of hyperautofluorescence were observed (**left**), but disappeared 12 weeks later without treatment (**right**). However, after resolution of hyperautofluorescence areas, scars (dark dots nasal to disc on right) were noted as compatible with MFC. Indeed, the patient developed a recurrence 2 years later and the diagnosis was reverted to MFC.

BAF is especially useful in choriocapillaritis entities, including MEWDS; APMPPE; idiopathic multifocal choroiditis (MFC); serpiginous choroiditis (SC), whether TB associated or not; and acute syphilitic posterior placoid chorioretinitis (ASPPC), as well as primary photoreceptoritis cases such as acute zonal occult outer retinopathy (AZOOR). In combination with OCT, BAF performs almost as well as ICGA in diagnosis, although less globally. It is equally useful for follow-up in conditions such as MEWDS, APMPPE and MFC, as it avoids repeated dye injections (Figure 2). The integrated use of these diverse imaging methods is essential for a proper diagnostic and therapeutic approach to uveitis.

## 2. Optical Coherence Tomography Angiography (OCT-A)

Unlike the other complementary imaging modalities, which were clearly contributive to the evaluation of certain ocular structures in uveitis entities, but were never considered nor pretended to replace the dye angiographic methods, OCT-A was put forward as being able to be a non-invasive method that could replace classical angiographic dye methods.

Without going into details as to a description of OCT-A, which has already been explained in many detailed articles [18], we will give a brief account of the method. OCT-A is an evolution of OCT technology. It uses multiple scans per second to detect differences between the images, and especially the movement of red blood cells, in order to provide anatomic information about the scanned area. The images are firstly captured in a 3D motive, which is reconstructed and presented to the clinician as 2D cross sectional and en face scans [19]. As the capture of images is essential to the quality of the provided information, motion artifacts due to movement of the patient, or mirroring of the superficial vessel to lower structures were the major problems of the OCT-A. Moreover, common clinical features of patients with uveitis, such as subretinal fluid (in particular in VKH), may be acting as significant cofounders, since the fluid itself may decrease the decorrelation signal detected by the OCT-A machine [20]. New software such as the projection artifact removal OCT-A (PAROCTA) helped with the decrease in motion artifacts [21]. The limited field of view was another major disadvantage when the exam was compared to dye exams. Furthermore, new devices such as the Xephilio are promising a wider field of view, which would extend to 20 × 23 mm [22]. The presentation of the retinal vasculature and choriocapillaris without the use of a dye was a revolution which led to theories that OCT-A could replace the dye exams. Even though its use in everyday routine practice is of limited value after other multimodal investigations have been performed (see below), it was the origin of remarkable research data in numerous studies.

Although OCT-A is a very high-quality research tool, its ability to replace dye angiography in current uveitis practice was recently questioned by our group, as we were concerned with the overamplification of its applications in current uveitis practice [23]. This pilot study motivated us to verify whether there was sufficient evidence in the literature to support these points (possible exclusive use of OCT-A and possible substitution to dye methods) or not, or whether our supposition of over-estimation of practical use of OCT-A, only based on cases so far, was supported by more sound proof in a literature search.

As a result, our purpose in this study was to use pioneering pragmatism, perform a literature search and analyze the aims and outcomes of OCT-A studies in uveitis, and then to list its most useful applications and analyze the arguments, if any, claiming or suggesting its effectiveness for global routine evaluation and follow-up of posterior uveitis as a substitute for dye angiographic methods.

## 3. Methods

A PubMed literature search was undertaken using the terms “OCT-A and uveitis”, “OCTA and uveitis” and “OCT-angiography and uveitis”. Articles were assigned to the following categories: (1) technical informative articles, (2) research articles and (3) review articles. Particular attention was given to articles claiming that OCT-A could replace dye angiographic methods in the appraisal and follow-up of uveitis. With this objective in view, special attention was applied to major review articles. Case report articles were excluded.

We further counted and listed the number of “OCT-A articles for uveitis” per year, starting in 2016. The articles gave information either on retinal circulation, choroidal circulation or both. We also listed the more frequent clinical entities investigated in these research articles.

## 4. Results

### 4.1. Number of Articles (2016–2022)

**Scheme 1:**
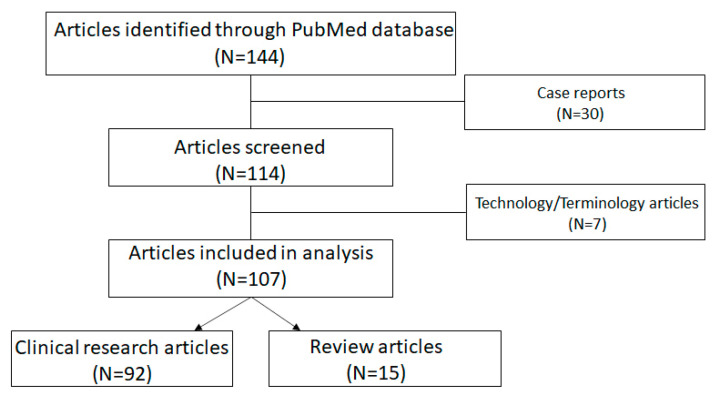
Article selection for systematic review using the terns “OCT-A and uveitis”, “OCTA and uveitis” and “OCT-angiography and uveitis”.

One hundred and forty-four articles including the aforementioned search terms were identified (Scheme 1). Case report articles were excluded, leaving 114 articles. We found that 4 articles were published in 2016, 17 in 2017, 14 in 2018, 21 in 2019, 14 in 2020, 18 in 2021 and 26 before the end of August 2022. After having been more or less constant from 2017 until 2021, there was a substantial increase in articles in 2022. Of the articles, 7 were mainly technical informative or consensus-based definition or standardization articles [22,23,24,25,26,27,28,29,30], 92 were research articles and 15 were review articles or articles directly comparing OCT-A to dye methods. The latter two categories were analyzed in more details in the next two sections.

### 4.2. Clinical Research Articles

The search yielded 92 research articles on different disease conditions and different localizations of structures. Of these articles, 32 concerned retinal circulation, 45 concerned choroidal circulation and 14 explored both retinal and choroidal circulation. Nineteen articles concerned the investigation of inflammatory choroidal neovascularization (CNV). Most articles indicated the adjunctive value rather than the exclusive use of OCT-A, but the positive contribution was the fact that evolution of CNV could be followed with reduced necessity of dye injection during treatment [31,32,33]. One article rated the detection of CNV by OCT-A as high as 76.9% compared to dye angiography [34]. Two articles focused on the fact that OCT-A was useful to distinguish CNV from inflammatory lesions [35,36]. Two articles found that OCT-A was insufficient to distinguish active from inactive CNV [37], and should, therefore, be used as part of multimodal imaging [38]. One article stated that OCT-A was not sufficient to replace dye angiography [39].

The most frequently analyzed conditions were the choriocapillaritis entities, including SC, TB-related or not, MFC/PIC, MEWDS and APMPPE (N = 35), Behçet’s disease (N= 15) and VKH disease (N = 8). This classification gives an idea for which diseases OCT-A is probably going to be most useful when it is integrated into the multimodal imaging investigation.

### 4.3. Review Articles

Fifteen review articles were reviews or touched upon the comparison of OCT-A and dye angiographic methods and directly or indirectly hinted at the possibility of OCT-A replacing dye angiographic methods, but failed to prove such a paradigm. We analyzed most of these articles for such a possibility.

Herbort and al. concluded that the claim of OCT-A to possibly replace classical dye angiographic methods for routine follow-up and management of posterior uveitis was largely overrated [18]. Similarly, Pichi and Hay declared that “OCT-A data and results were of limited utility to the ophthalmologists who are looking to apply OCTA in their everyday uveitis clinic” [40]. On the other hand, this review outlined most of the inflammatory situations where OCT-A represented a precious adjunct to classical angiographic investigation. The authors further put forward that OCT-A could replace ICGA in detecting choroidal granulomas, a statement which should be considered cautiously for everyday clinical practice while taking into account the still-limited field of view of routinely used instruments. Since its availability, probably because of the high technicity of the OCT-A method, the unsubstantiated notion that OCT-A could replace the global use of dye methods in uveitis has been circulating and up in the air. This speculation was the origin of several articles attempting to support such a postulate. This approach stands out in a recent article by multiple authors entitled “Experts opinion: OCTA vs. FFA/ICG in uveitis-which will survive?” [41]. Even though the conclusion of this article was not in favor of OCT-A, multiplying such articles tends to falsely strengthen the idea of a possible replacement of dye angiographic methods. Furthermore, it is not suitable to oppose these two methods, as in reality, they are complementary. Another article attempted to compare ICGA and OCT-A in choroiditis, with the aim to show that the latter was able to replace the former method [42]. Indeed, beyond the articles merely hinting that OCT-A represented more than just a complementary imaging modality such as OCT, EDI-OCT and FAF, some articles attempted to indicate that OCT-A was able to replace classical angiographic modalities in the appraisal and monitoring of uveitis [40,42]. Although this was not the official purpose of their article, Tian et al. [42], by trying to imply that OCT-A could possibly be compared to ICGA in posterior uveitis and putting them on an equal footing, precisely leads the reader to believe that one method could replace the other [42]. Besides the many drawbacks of OCT-A mentioned by the article itself, the article was characterized by several flaws. The authors considered the terms of uveitis and choroiditis as equivalent. Indeed, it is well-known that ICGA merely gives information on the choroid and not on other structures, and these terms are not equivalent. To declare that “posterior uveitis is also termed choroiditis” is a blatant misconception. Therefore, the inclusion of Behçet’s uveitis cases in their collective is not justified, as it is a purely retinal disease and does not present choroidal ICGA lesions. As far as technical matters are concerned, OCT-A montage was used to obtain a wider field of vision. A disadvantage of a montage is the presence of artifacts including, but not limited to, displacement, shadowing and vessel displacement. These can decrease the quality of the images and, as a consequence, the quality of the diagnosis and follow-up. On the contrary, ICGA covers a greater field of view with a better quality of imaging without the need of a montage. Additionally, a single ICGA time frame was used for comparison to OCT-A. It is well-known that ICGA is a dynamic exam which can provide information throughout the intermediate and late phases about the localization of the inflammation (choriocapillaris, full stroma of choroid or part of the choroidal stroma). According to the results of their study, in 64% of cases where hypofluorescence was shown on ICGA, OCT-A was unable to demonstrate these areas as they were outside the field of vision. OCT-A also failed to show which mechanism was at the origin of drop-out areas, choriocapillaritis or stromal choroiditis, which a dynamic analysis of ICGA frames is able to demonstrate. In conclusion, Tian et al. presented a case series of patients with posterior uveitis (in fact, choroiditis) where ICGA was superior to OCT-A in analyzing choroidal inflammation, failing to put forward OCT-A as a credible alternative to ICGA. At this time and with the current OCT-A technology routinely available to eye centers, ICGA remains the gold standard exam in the diagnosis and follow-up of choroidal inflammation. An article on the use of OCT-A in “so-called” white dot syndromes insisted on the complimentary information obtained with this imaging modality without claiming that it could replace classical dye-based angiography [43]. In their article, Invernizzi et al. stressed the importance of a multimodal approach to imaging of choroiditis, including ICGA and OCT angiography without establishing a hierarchy or precedence of one modality over the other [44]. Tranos et al. also compared OCT-A to FA and ICGA. Like many other review articles, it described the aspects of uveitis in which OCT-A was useful and pointed out the many complementary advantages it represented in addition to FA/ICGA, without going as far as to consider it as a possible exclusive angiographic modality [45]. Similarly to other reviews, Dingerkus et al. exposed situations where OCT-A was especially useful [46]. However, like all other reviews, their conclusion, considering the precious information yielded by OCT-A, was that “it should be used as a complementary modality rather than as an exclusive tool”. An earlier review by Invernizzi et al. insisted on the fact that OCT/OCT-A are “becoming more essential in the management of uveitis”; however, they did not put them in perspective with dye angiographic methods and refrained from any statement on the possibility that they might replace the latter [47]. The most complete and technically detailed review of OCT-A was published in 2018 by Spaide et al. [19]. As far as uveitis is concerned, the article addresses mainly general vasculitis aspects, not going into illustrations of practical disease. At this early stage of development, the authors state that “none of the older imaging modalities can be replaced by OCT-A at present”. Kashani et al. laid down the foundations and arguments of the situations where classical dye methods or OCT-A were more appropriate to be used in uveitis, a topic which will be discussed in another section of this analysis [48]. These authors mainly discussed the advantages of OCT-A in retinal/macular inflammatory diseases. In the comprehensive early review of Pichi et al. on OCT-A in uveitis, its applications in uveitic conditions are illustrated, but the authors conclude that “in its current state, OCTA serves as a useful adjunct to traditional angiographic techniques, since multimodal image analysis is still necessary to confirm pathological findings in uveitis” [49]. In this almost exhaustive group of review articles analyzing OCT-A starting in 2017, no article whatsoever claims nor hints that OCT-A is in a position to replace conventional dye angiographic methods. It must be considered, like OCT, EDI-OCT and BAF, as an adjunct/complementary imaging method, to be integrated in the multi-modal imaging of uveitis, although it is of high technical value.

### 4.4. Significant Contribution of OCT-A to Uveitis

**Figure 3:**
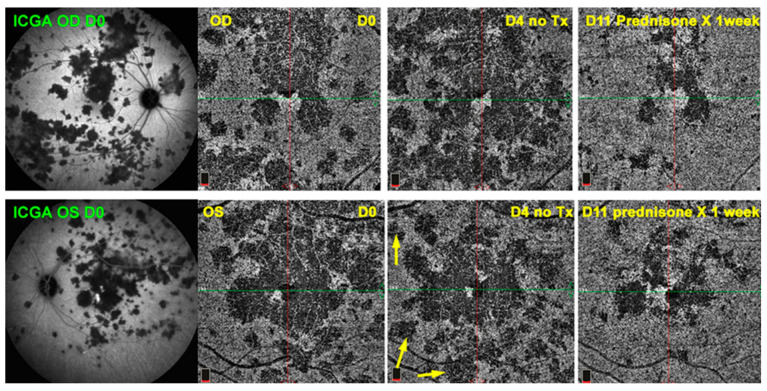
OCT-A follow-up of APMPPE. Example of practical usefulness of OCT-A in a patient with APMPPE (same patient as Figure 1). At presentation, ICGA frames clearly establish the global involvement of the fundus. To monitor the evolution of lesions and the impact of therapy, OCT-A allowed a close follow-up, although only of the central lesions (6 frames to the right). Yellow arrows show new areas of drop-out before regression of lesions following introduction of corticosteroids (frames at the extreme right).

There is no doubt that OCT-A represents significant progress in the appraisal of uveitis, as evidenced by the many research articles published since the method became available. We will refrain from citing again what has been extensively and repeatedly described. Many of these studies report new data which is still to be standardized in order to possibly be applied to everyday routine practice. Furthermore, the experiments were often performed on research instruments not readily available to general clinicians. We will, therefore, focus on the major intakes immediately beneficial for the practitioner in the daily management of patients. When investigating this point, we reviewed the utility of OCT-A in a specific center that uses a routinely available OCT-A instrument and evaluated, using pioneering pragmatism, the situations in which OCT-A provided further determining information to dye angiography, OCT, EDI-OCT and BAF data. Additionally, practical use reported in the literature was also considered. As a general rule, OCT-A was performed in most patients consulting for uveitis at presentation and for follow-up in the center. Within the 217 new uveitis cases seen from 2018 to 2022 in this center, 146 were examined with an Optovue instrument and 46 posterior uveitis cases were analyzed. Rare were the cases for which OCT-A was of crucial additional utility in the practical management of patients, so they will not be individually described hereafter. It did not provide diagnostic help in any patient after multimodal imaging was performed. It was useful for follow-up in two cases of APMPPE, showing the evolution of choriocapillary drop-out without the need to repeat ICGA (Figure 3). In five cases of inflammatory CNV due to MFC and APMPPE, it also helped with monitoring the evolution during treatment and allowed the researchers to reduce the number of dye angiographies performed.

The main situations where OCT-A was of determining utility in everyday practice from the literature were the rare inability to use fluorescein dye and the even rarer inability to use ICG because of allergic reactions. OCT-A was shown to be useful and to help in the detection of CNV, such as in MFC/PIC and other choriocapillaritis entities as well as its follow-up during treatment, but none of the articles recommended it for exclusive use. [31] One article clearly stated that OCT-A was not sufficient to replace dye angiography [39], and two articles indicated that OCT-A was unable to distinguish active from inactive CNV [37,38]. In predominantly retinal diseases, such as Behçet’s uveitis, OCT-A cannot be considered as essential in the diagnosis. However, analysis of macular microcirculation and its monitoring have shown to be useful to the clinician at times, although no standardization has been put forward so far [50]. The most useful application of OCT-A was for the group of choriocapillaritis diseases, including APMPPE, MFC and SC, and its utility resided mainly in the close follow-up it allowed, as no dye injection was needed [51] (Figure 3). As far as MEWDS is concerned, OCT-A was useless, as end-capillary low flow circulation is not identified by OCT-A. This was the origin of the erroneous thinking that there was an absence of choriocapillaris non-perfusion and, thus, an alleged intact choriocapillaris, as well as that MEWDS was supposedly a primary photoreceptoritis [52]. Although it was not included in our search terms, when trying to establish the essential, practical contributions of OCT-A, the article of Abucham-Neto et al. should be cited [53]. These authors indicate that OCT-A may better identify new vessels obscured by retinal hemorrhage, early peripapillary neovascular proliferation and telangiectasias. In this article, they also wrote “OCT-A was not able to detect clear signs of active inflammation around the affected vessels”.

Finally, among the anecdotal descriptions of cases that benefited from OCT-A, we would like to mention the report of suspected retinal granulomas in a sarcoidosis case [54]. This indicates that, despite the limited practical use of OCT-A, it is worthwhile to perform the test, as unexpected findings can occur.

## 5. Discussion

Since 2016, more than 150 articles have been published on OCT-A in PubMed, the only database we considered in this search. This list included case reports, research articles, technical articles and reviews. The fact that this imaging modality gives information on the retinal and choroidal circulation without the need for dye injection raised great hope that it could replace conventional angiographic dye methods. The amount of new data that became available on uveitis through all these publications generated the assumption that this might be the case. Proof of such a belief were several articles putting into competition the two angiographic modalities, such as a recent article entitled “Experts opinion: OCTA vs. FFA/ICG in uveitis-which will survive?” [41]. The two crucial questions to which we were seeking answers through this search were whether the possibility of OCT-A replacing dye angiographic methods was a reality, and we linked to it the auxiliary question of whether it was an alternative for everyday clinical practice. Analyzing several retinal and choroidal inflammatory disorders, we concluded that the role of OCT-A had been largely overestimated as a routine follow-up tool for the management of posterior uveitis, including choroiditis, for which it was particularly ill-suited [18]. To go further and investigate whether routine use of OCT-A was suited for everyday diagnosis and follow-up in uveitis in place of conventional dye angiography, or whether this represented a mystification, we conducted an extensive literature review in search of arguments in one direction or the other. Looking especially at review articles or articles comparing the two modalities, we did not find a single article suggesting that OCT-A could be a substitute to conventional dye angiography, let alone to prove this. All of these articles indicated that, like other new imaging modalities, OCT-A was a valuable complementary tool to be integrated into multimodal imaging rather than being considered as a substitute. The reason why OCT-A was given a more preeminent status in comparison to other imaging modalities, and why it reached such an aura, is interesting to consider. Firstly, OCT-A is indeed a technical high-grade imaging modality with spectacular research results not necessarily applicable to daily ophthalmological consultation, but also subject to overinterpretation. Further, a majority of the research articles came from institutions with access to research instruments and which are interconnected through shared authorships, representing a common line of thought. The large proportion of high-quality research articles coming from the same limited, very active groups working in close contact with instrument companies has possibly contributed to artificial inflation of the practical importance of OCT-A. These very productive groups, in their repeated articles, tend also to overemphasize their conclusions, with a trend of demonstrating useful daily use. Indeed, when promoting an instrument, it is commercially more interesting to affix the label of routine everyday use rather than that of being predominantly an instrument of research purposes.

Taking into consideration that the two dye exams are held at the same time, the benefits of these exams are that the clinician can find information about filling details of the retinal or choriocapillaris/choroidal circulation (delayed filling, non-perfusion). As dye exams offer a dynamic analysis blood flow and flow of liquids, the permeability of the inner retinal barrier (presence of leakage or not) and of the outer retinal barrier (leakage through the RPE) can be investigated. Additionally, ICGA precisely detects the presence of inflammatory foci in the choroidal stroma, identified by the flow voids to the ICG molecule they create. Moreover, the aforementioned pieces of information are not limited to the posterior pole. They summarize the advantages of the dye exams over OCT-A in the daily routine practice of a uveitis clinic. Table 1 summarizes the major advantages/disadvantages of FA/ICGA versus OCTA in routine uveitis practice.

In summary, considering the strong present trend promoting OCT-A, it should be mentioned that it could be deleterious to convey the illusion to the clinician that OCT-A is capable of replacing the global investigation obtained with dual FA/ICGA, or to propagate the belief that these methods can be abandoned in favor of OCT-A. The latter is unable to furnish the precious global information available through dye angiography. Furthermore, OCT-A fails to visualize the breakdown of the blood–retina barrier, an essential biomarker in the assessment of retinal vasculitis in uveitis. Taking into account the caveats mentioned in our study, the limitations for practical clinical use and the lack of standardization for potential routine use, the complementary information obtained through OCT-A is, however, of high-grade value. When not considered as an exclusive modality in uveitis, there is no doubt that OCT-A should be considered as an example of substantial progress in uveitis research. It should be integrated, like all other complementary imaging methods cited, in the modern multimodal appraisal of uveitis.

## Figures and Tables

**Table 1:** Fluorescein angiography vs. ICGA angiography vs. OCT-A angiography.

Fluorescein Angiography	ICG Angiography	OCT-Angiography
Invasive	Invasive	Non-invasive
Widefield analysis	Widefield analysis	Current technology offers limited field of view in commercially available machines
Kinetic analysis of blood flow and flow of liquids	Kinetic analysis of blood flow and flow of liquids	Static blood vessel imaging (sufficient flow necessary to detect vessels)
Information on the retina (choriocapillaris on the first 40–60 s)	Information on the choroid (some vascular information about the retina)	Information on the retina and choriocapillaris
Side effect from dye (rare)	Side effect from dye (very rare)	No dye needed
Non-repeatable at short interval, ±10 min exam duration	Non-repeatable at short interval, ±30 min exam duration	Repeatable, <5 min capture
Should be avoided in pregnancy or renal dysfunction (or performed with caution)	Should be avoided in pregnancy or hepatic dysfunction (or performed with caution)	No limitations
Information on retinal vascular leakage	Information on choroidal vascular leakage	No information on vascular leakage
Information on active iCNV	Information on active iCNV	Detection of iCNV (distinction between active/non-active not always available)
Dual FA/ICGA ➔ Global information on all inflammatory diseases two-dimensional	Dual FA/ICGA ➔ Global information on all inflammatory diseases two-dimensional	Limited information on limited disease entities three-dimensional

ICG: indocyanine green; OCT: optical coherence tomography; ILM: internal limiting membrane RPE: retinal pigment epithelium; iCNV: inflammatory choroidal neovascularization.

## Data Availability

Please address to the corresponding author.
